# The Effects of *swnN* Gene Function of Endophytic Fungus *Alternaria oxytropis* OW 7.8 on Its Swainsonine Biosynthesis

**DOI:** 10.3390/ijms251910310

**Published:** 2024-09-25

**Authors:** Chang Liu, Ning Ding, Ping Lu, Bo Yuan, Yuling Li, Kai Jiang

**Affiliations:** 1 College of Life Science and Technology, Inner Mongolia Normal University, Hohhot 010022, China; ecibxivq@outlook.com (C.L.); ndingning@163.com (N.D.); yuanbo934@126.com (B.Y.); liyuling@163.com (Y.L.); jiangkai@imnu.edu.cn (K.J.); 2 Key Laboratory of Biodiversity Conservation and Sustainable Utilization in Mongolian Plateau for College and University of Inner Mongolia Autonomous Region, Hohhot 010022, China

**Keywords:** SW, gene knockout, *A. oxytropis* OW 7.8, *swnN* gene

## Abstract

The *swnN* gene in the endophytic fungus *Alternaria oxytropis* OW 7.8 isolated from *Oxytropis glabra* was identified, and the gene knockout mutant Δ*swnN* was first constructed in this study. Compared with *A. oxytropis* OW 7.8, the Δ*swnN* mutant exhibited altered colony and mycelia morphology, slower growth rate, and no swainsonine (SW) in mycelia. SW was detected in the gene function complementation strain Δ*swnN*/*swnN*, indicating that the function of the *swnN* gene promoted SW biosynthesis. Six differentially expressed genes (DEGs) closely associated with SW synthesis were identified by transcriptomic analysis of *A. oxytropis* OW 7.8 and Δ*swnN*, with *P5CR*, *swnR*, *swnK*, *swnH2*, and *swnH1* down-regulating, and *sac* up-regulating. The expression levels of the six genes were consistent with the transcriptomic analysis results. Five differential metabolites (DEMs) closely associated with SW synthesis were identified by metabolomic analysis, with *L*-glutamate, α-ketoglutaric acid, and *L*-proline up-regulating, and phosphatidic acid (PA) and 2-aminoadipic acid down-regulating. The SW biosynthetic pathways in *A. oxytropis* OW 7.8 were predicted and refined. The results lay the foundation for in-depth elucidation of molecular mechanisms and the SW synthesis pathway in fungi. They are also of importance for the prevention of locoism in livestock, the control and utilization of locoweeds, and the protection and sustainable development of grassland ecosystems.

## 1. Introduction

Locoweeds primarily refer to the toxic plants of the genera *Astragalus* and *Oxytropis* that contain swainsonine (SW) [1,2]. These plants are widely distributed across arid and semi-arid grasslands around the world, including countries in the Northern Hemisphere such as the United States, Russia, and China, as well as some countries in the Southern Hemisphere including Australia, New Zealand, and Brazil [3,4,5,6]. Consumption of locoweeds by livestock results in locoism, with symptoms including neurological disorders, dysgenesis, emaciation, addiction [7,8,9], and even death in severe cases, which cause considerable losses for grassland animal husbandry [10,11,12,13,14,15].

The SW-producing locoweed endophytic fungi were first isolated from *O. sericea*, *O. lambertii*, and *A. mollisimus*, and were identified as *Embellisia* 16] and revised to *Alternaria* later [16,17,18,19]. An SW-producing endophytic fungus was isolated from *O. glabra* by our research group, and SW was not detected in the plants without this endophytic fungus. The fungus was identified as *Alternaria* by analysis of morphological and DNA sequence. Therefore, we proposed that the toxicity of *O. glabra* was caused by its endophytic fungus *Alternaria oxytropis* [20,21]. Previous reports proposed that SW was synthesized by endophytic fungi in locoweeds [16,22].

SW is an indolizidine alkaloid [23] initially isolated and purified from *Swainsona canescens* and other plants including locoweeds and *Ipomoea* [24,25,26,27,28]. SW competitively inhibits the activity of lysosomal α-mannosidase I and Golgi α-mannosidase II, leading to inhibition of glycoprotein synthesis and the accumulation of mannose within cells, which causes cellular vacuolar degeneration and results in metabolic dysfunction in animals [9,10,11,23,24].

The *saccharopine reductases* (*sac*) gene (KY052048) was previously knocked out in *A. oxytropis* OW 7.8 isolated from *O. glabra* by our research group. Compared with *A. oxytropis* OW 7.8, *sac* gene knockout mutant M1 exhibited decreased levels of SW and saccharopine while the level of *L*-lysine did not change significantly [29]. The SW level was higher in the *sac* gene complementation strain C1 than in *A. oxytropis* OW 7.8 and M1 [30], suggesting that the function of the *sac* gene promoted SW synthesis in fungi.

A comparative genomic analysis was conducted on *Metarhizium robertsii*, *Ipomoea carnea* endophyte, *Arthroderma otea*, *Trichophyton equinum*, *A. oxytropis*, and *Pseudogymnoascus* sp., suggesting the presence of an “SWN gene cluster” closely related to SW synthesis in these fungi [31]. The SWN gene cluster consists of *swnA*, *swnH1*, *swnH2*, *swnK*, *swnN*, *swnR*, and *swnT* (Table 1). However, the *swnA* and *swnT* genes do not exist in all SW-producing fungi. For example, there are no *swnA* and *swnT* genes in *A. oxytropis* and *Pyrenophora semeniperda*. *Slafractonia leguminicola* lacks the *swnA* gene, and *Chaetothyriaceae* sp. does not have the *swnT* gene [32].

The *swnN* gene encodes one of the reductases in the Rossmann-fold family [31]. SW levels decreased significantly when the *swnN* was knocked out in *M. robertsii* with PA levels significantly increasing, and the 1-oxoindolizine levels were higher than that in the *swnR* gene knocked out strain Δ*swnR* and the *swnN*/*swnR* double knockout strain Δ*swnNR*. The SwnN protein was hypothesized to catalyze the reduction of 1-hydroxyindolizine from 1-oxoindolizine [33].

**Figure 1:**
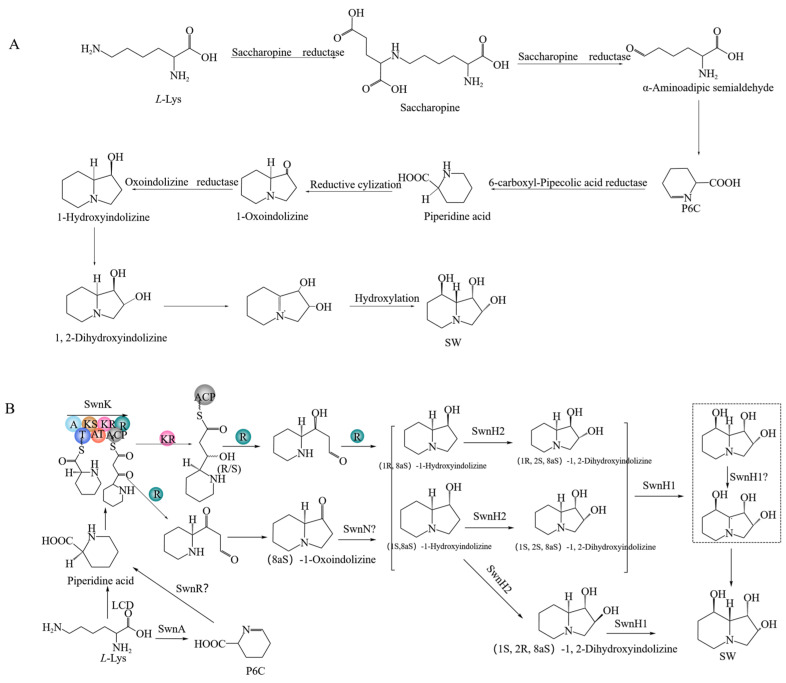
SW synthesis pathways in two fungi (**A**): *S. leguminicola*, (**B**): *M. robertsii*.

Research on SW biosynthesis pathway in fungi began with *Slafractonia* (formerly Rhizoctonia) *leguminicola* [34], followed by in *M. robertsii* and *A. oxytropis* [35,36,37]. The predicted SW biosynthesis pathway in *S. leguminicola* is as follows [38,39]: *L*-lysine → saccharopine → α-aminoadipic semialdehyde → P6C → *L*-PA → 1-oxoindolizidine → 1-hydroxyindolizine → 1,2-dihydroxyindolizine → SW (Figure 1A). The predicted SW biosynthesis pathway from *L*-lysine to *L*-PA has two branches in *M. robertsii* [31,33]: *L*-lysine → *L*-PA and *L*-lysine → P6C → *L*-PA. From *L*-PA to SW, there are also two branches: *L*-PA → 1-oxoindolizidine → 1-hydroxyindolizine → 1,2-dihydroxyindolizine → SW and *L*-PA → 1-hydroxyindolizine → 1,2-dihydroxyindolizine → SW (Figure 1B). The predicted SW biosynthesis pathway from *L*-lysine to L-PA has two branches in *A. oxytropis* [36]: *L*-lysine ↔ saccharopine → α-aminoadipic semialdehyde → P6C → *L*-PA and *L*-lysine → 6-amino-2-oxohexanoate → P2C → *L*-PA. The process from *L*-PA to SW is predicted to be *L*-PA → 1-hydroxyindolizine → SW.

In this study, the *swnN* gene was first cloned and knocked out in *A. oxytropis* OW 7.8, and the Δ*swnN*/*swnN* gene complementation test was performed. The SW levels in the mycelia of the three strains were determined. Additionally, the analyses of transcriptome and metabolome were conducted on *A. oxytropis* OW 7.8 and Δ*swnN* to predict the SW synthesis pathway. The results lay the foundation for in-depth analysis of the molecular mechanisms and metabolic pathways of SW synthesis in fungi, and provide reference for future control of SW in locoweed which will benefit the development of grassland animal husbandry and the sustainable use of grassland ecosystem.

## 2. Results

### 2.1. Cloning and Bioinformatics Analysis of the swnN Gene in A. oxytropis OW 7.8

The *swnN* gene (GeneBank: OR596336) in *A. oxytropis* OW 7.8 was cloned, with a length of 1164 bp (ATG-TGA) and three introns (57 bp, 107–163 bp; 56 bp, 402–457 bp; 55 bp, 815–869 bp). The length of *swnN* cDNA is 996 bp (ATG-TGA). The *swnN* gene is predicted to encode a protein with 331 amino acids. The molecular formula of this protein is C_1646_H_2600_N_440_O_486_S_9_, with a molecular weight of 36.62 kDa and a pI of 5.49. The predicted protein is hydrophilic, without transmembrane domains.

**Figure 2:**
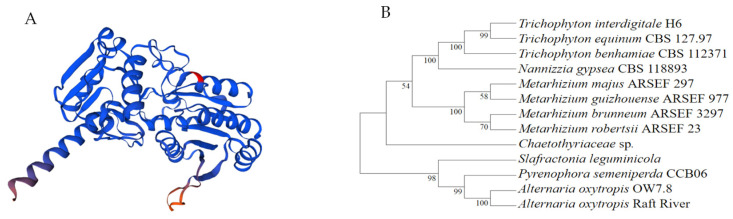
Bioinformatics analysis of the SwnN protein. (**A**): Predicted SwnN protein structure; (**B**): The phylogenetic tree of the SwnN protein.

A phylogenetic tree of SwnN proteins from 13 SW-producing fungi was constructed using the neighbor-joining method (Figure 2B) with two branches: The upper branch consisted of three species of the genus *Trichophyton* and one species of the genus *Nannizzia*, and another consists of four *Metarhizium* species and a separated branch of *Chaetothyriaceae* fungi. In the lower branch, *A. oxytropis* Raft River and *A. oxytropis* OW 7.8 first clustered together, then joined with *P. semeniperda*, and finally clustered with the separately branched *S. leguminicola*. The amino acid sequence alignment of SwnN proteins showed the highest sequence identity of 100% between *A. oxytropis* OW 7.8 and *A. oxytropis* Raft River. The sequence identity is 87.39% between *A. oxytropis* OW 7.8 and *S. leguminicola*, while 85.71% between *A. oxytropis* OW 7.8 and *M. acridum*, *Microsporum canis*, 84.03% between *A. oxytropis* OW 7.8 and three *Metarhizium* species (*M. brunneum* ARSEF 3297, *M. guizhouense* ARSEF 977, *M. robertsii* ARSEF 2575), approximately.

### 2.2. Sensitivity Screening of A. oxytropis OW 7.8 to Hyg B

After incubation in darkness at 25 °C for 20 days, the growth of *A. oxytropis* OW 7.8 colonies on PDA media containing different concentrations of hygromycin B (Hyg B) indicated that the fungus is sensitive to ≥2 μg/mL of Hyg B.

### 2.3. Identification for Transformants of ΔswnN Colonies

**Figure 3:**
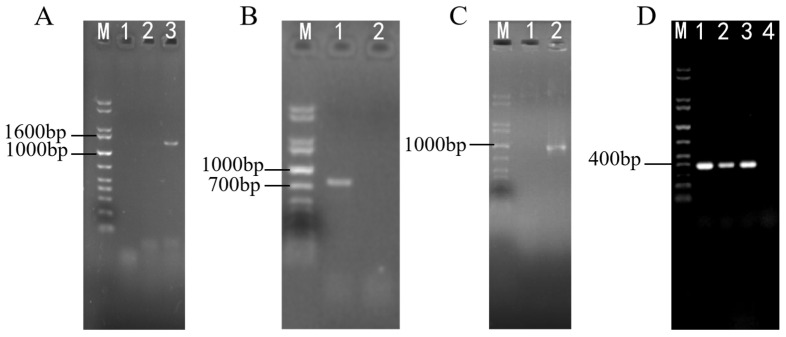
Electrophoresis analysis of PCR products of transformant DNA. Marker: 1 kb plus DNA Ladder. (**A**): Lanes 1, 2: negative control, Lane 3 shows a band of the *hpt* gene, with the expected product being 1388 bp. (**B**): Lane 1 shows a band of the upstream homologous sequence of *swnN* + *hpt* gene, with the expected product being 861 bp, Line 2: negative control. (**C**): Line 1: negative control, Lane 2 shows a band of the *hpt* gene + downstream homologous sequence of the *swnN*, with the expected product being 1055 bp. (**D**): Lane 1, 2, 3 show bands of the internal sequence of *swnN* with the expected product being 401 bp, Lanes 4: PCR results using *swnN* knockout transformant DNA as a template with no band.

The *swnN* gene knockout transformants gradually appeared on PDA media containing 2 μg/mL Hyg B one week after transformation. PCR results showed that bands of *hygromycin phosphotransferase* (*hpt*) gene, *hpt* gene and upstream homologous to the *swnN*, the *hpt* gene and downstream homologous to the *swnN* were amplified in *swnN* gene knockout transformants (Figure 3). The sequencing of the PCR products confirmed their accuracy, resulting in the identification of the ∆*swnN*.

### 2.4. Morphology of Colonies and Mycelia

**Figure 4:**
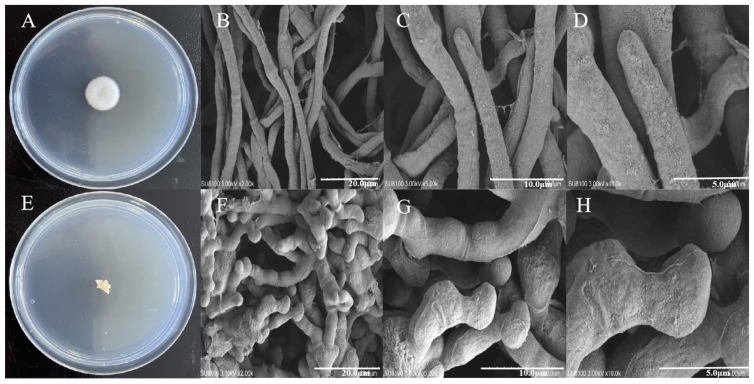
Morphology of colonies and mycelia from *A. oxytropis* OW 7.8 and Δ*swnN*. (**A**): *A.oxytropis* OW 7.8 colonies. (**B**–**D**): *A.oxytropis* OW 7.8 mycelia magnified 2000×, 5000×, and 10,000×. (**E**): Δ*swnN* colonies. (**F**–**H**): Δ*swnN* mycelia magnified 2000×, 5000×, and 10,000×.

The colonies of *A. oxytropis* OW 7.8 displayed soft white velvety patches, round, raised, with uniform margin and radial growth colonies which grew slowly (Figure 4A). Later a black brown pigment was secreted in each colony [21]. In contrast, the colonies of ∆*swnN* were loose creamy yellow and irregularly shaped, with no pigment accumulation and slower growth rate (Figure 4E).

Significant differences were observed in the mycelial morphology and structure between *A. oxytropis* OW 7.8 and Δ*swnN* with scanning electron microscopy. *A. oxytropis* OW 7.8 exhibited typical single, tubular, smooth-surfaced and unbranched fungal hyphae (Figure 4B–D). In contrast, Δ*swnN* showed abnormal jointed hyphae, with protrusions, depressions, and swollen branched tips (Figure 4F–H).

### 2.5. Screening of ΔswnN for Glufosinate Sensitivity

After incubation in darkness at 25 °C for 20 days, the growth of Δ*swnN* colonies on PDA media containing different concentrations of glufosinate (Gla) indicated that the fungus is sensitive to ≥500 μg/mL of Gla.

### 2.6. Screening and Identification of ΔswnN/swnN

**Figure 5:**
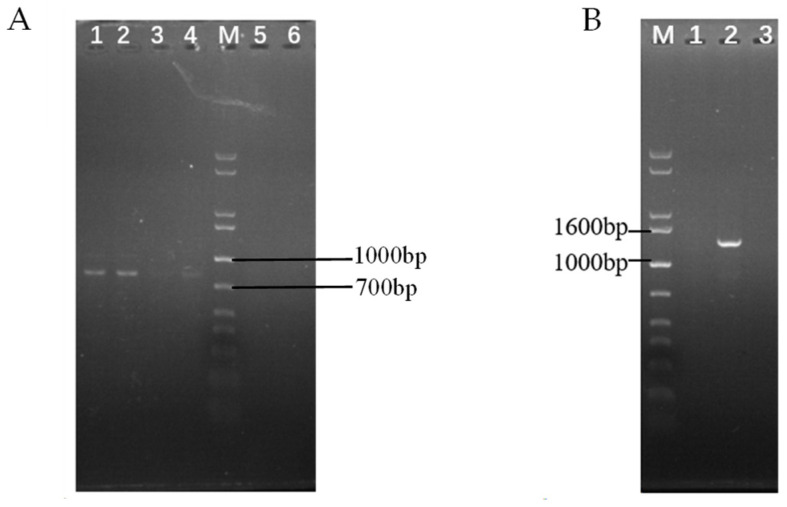
Electrophoresis analysis of PCR products of *swnN* gene function complement transformants DNA. Marker: 1 kb plus DNA Ladder. (**A**): Lanes 1, 2, 3, and 4 show bands of the *bar* gene, with the expected product being 816 bp, Line 5, 6: negative control. (**B**): Lane 2 shows a band of the *swnN* cDNA, with the expected product being 1356 bp, Line 1, 3: negative control.

PCR results showed that sequence of the glufosinate resistance (*bar*) gene and the *swnN* cDNA were amplified in the *swnN* gene function complement transformants (Figure 5). The sequencing of the PCR products confirmed their accuracy, resulting in the verification of the gene function complement strain Δ*swnN*/*swnN*.

### 2.7. SW Levels in A. oxytropis OW 7.8, ΔswnN, and ΔswnN/swnN Mycelia

**Figure 6:**
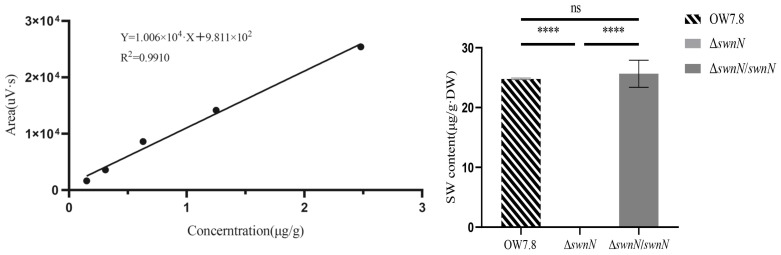
SW level on day 20 in *A. oxytropis* OW 7.8, Δ*swnN* and Δ*swnN*/*swnN*. Error bars represent the standard error of the mean (*n* = 3), (****) *p* < 0.0001. ns: not significant.

The regression equation for the SW standard curve is Y = 1.006 × 10^4^X + 9.811 × 10^2^ (R^2^ = 0.9910). SW was not detected in the mycelia of Δ*swnN*, whereas the SW levels in the mycelia of *A. oxytropis* OW 7.8 and Δ*swnN*/*swnN* were 24.799 ± 0.132 μg/g·DW and 25.656 ± 2.258 μg/g·DW after 20 days of cultivation (Figure 6), indicating that the gene function of *swnN* promotes SW synthesis.

### 2.8. Transcriptome Sequencing Analysis of A. oxytropis OW 7.8 and ΔswnN

**Figure 7:**
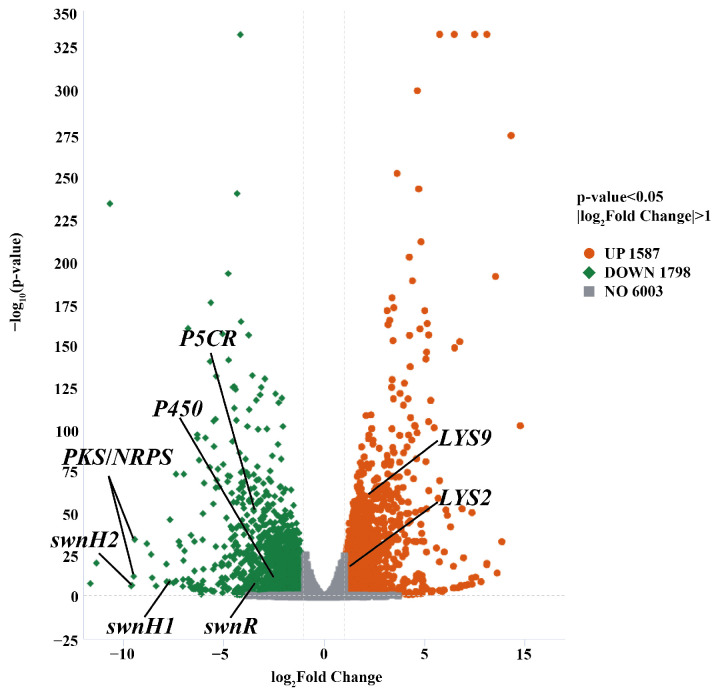
Volcano plot of DEGs between *A. oxytropis* OW 7.8 and Δ*swnN*.

There was no *swnN* transcripts detected from Δ*swnN* transcriptomics. Transcriptome sequencing of *A. oxytropis* OW 7.8 and Δ*swnN* produced a total of 254,788,656 clean reads, amounting to 38.21 G. Gene differential expression was shown between *A. oxytropis* OW 7.8 and Δ*swnN*. A total of 3385 DEGs were identified, of which 1587 (46.89% of DEGs) were up-regulated and 1798 (53.11%) were down-regulated. Five DEGs closely related to SW synthesis were identified, among which the gene of sac was up-regulated, while the genes of *swnR*, *swnK*, *swnH1*, and *swnH2* were down-regulated (Figure 7).

**Figure 8:**
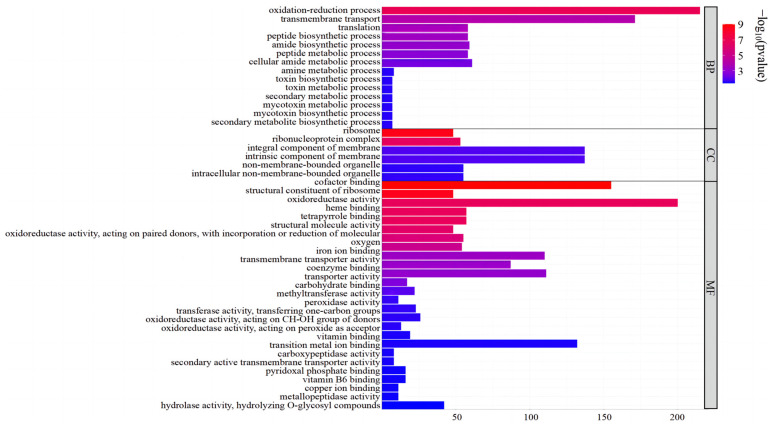
GO annotation of DEGs between *A. oxytropis* OW 7.8 and Δ*swnN*.

GO functional enrichment analysis of DEGs between *A. oxytropis* OW 7.8 and Δ*swnN* showed that 701 DEGs (277 up-regulated and 424 down-regulated) were assigned to 47 GO terms. In the Biological Process (BP) category, the top three enriched metabolic groups were oxidation–reduction process (30.67%), transmembrane transport (24.39%), and cellular amide metabolic process (8.70%). In the Cellular Component (CC) category, the top three groups were membrane component (19.54%), intrinsic component of membrane (19.54%), and non-membrane-bounded organelle (7.85%). In the Molecular Function (MF) category, the top three groups were oxidoreductase activity (28.53%), cofactor binding (22.11%), and transition metal ion binding (18.83%) (Figure 8).

**Figure 9:**
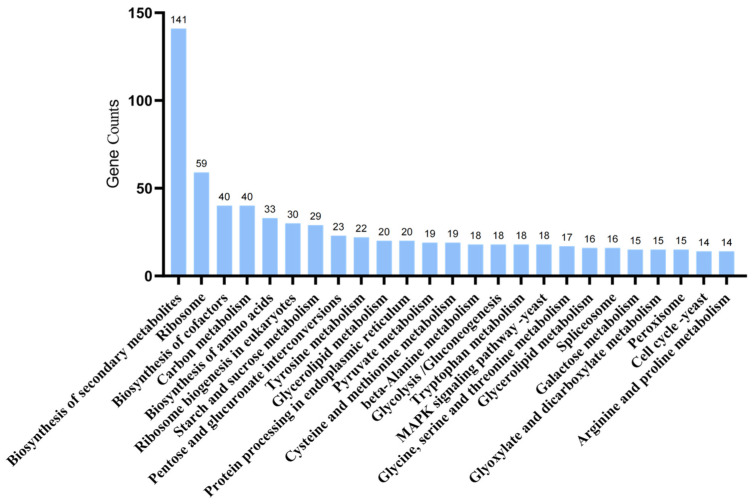
KEGG enrichment analysis of DEGs between *A. oxytropis* OW 7.8 and Δ*swnN*.

KEGG enrichment analysis of DEGs between *A. oxytropis* OW 7.8 and Δ*swnN* annotated 553 DEGs (301 up-regulated and 252 down-regulated) to 97 pathways. The top three metabolic groups (Figure 9) included biosynthesis of secondary metabolites (25.50%), ribosome (10.04%), biosynthesis of cofactors (7.23%), and carbon metabolism (7.23%).

### 2.9. Metabolomic Analysis of A. oxytropis OW 7.8 and ΔswnN

**Figure 10:**
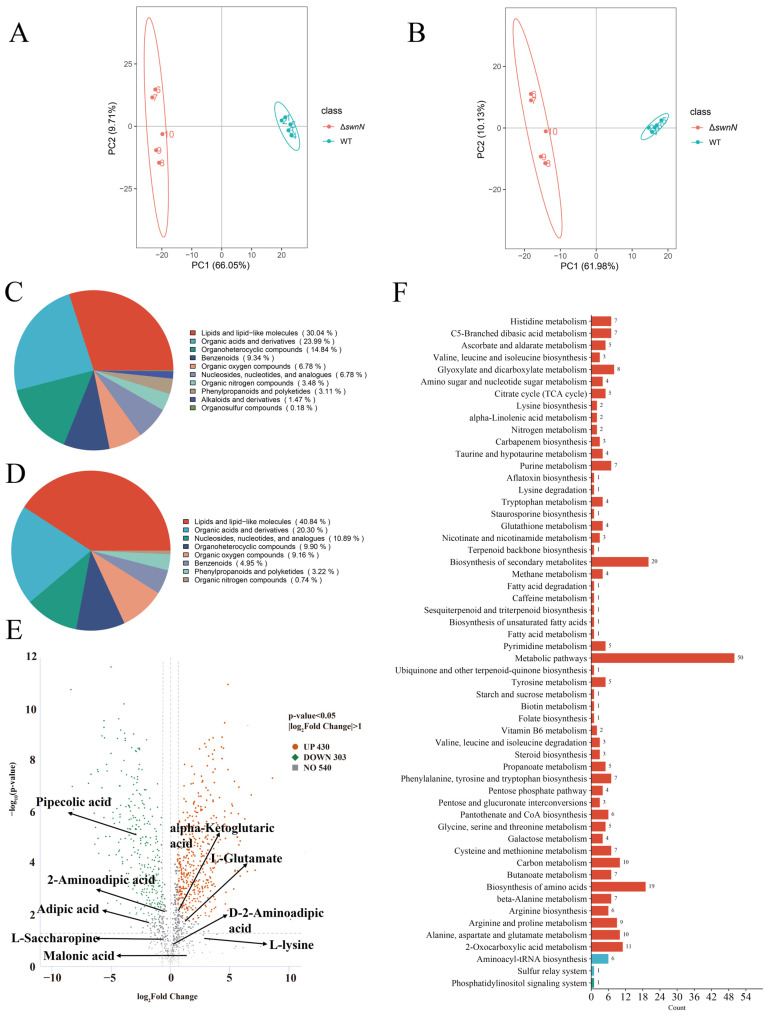

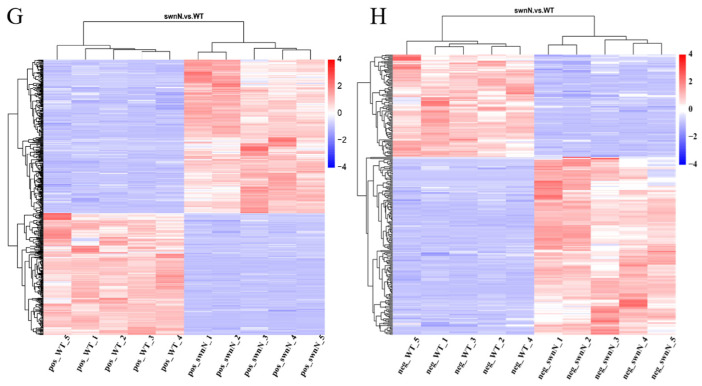
Metabolomic analysis of *A. oxytropis* OW 7.8 and Δ*swnN*. (**A**): Principal component analysis in positive ion mode. (**B**): Principal component analysis in negative ion mode. (**C**): Pie chart of metabolite classification in positive ion mode. (**D**): Pie chart of metabolite classification in negative ion mode. (**E**): Volcano plot of DEMs between *A. oxytropis* OW 7.8 and Δ*swnN*. (**F**): KEGG enrichment analysis of DEMs. (**G**): Heatmap of clustering analysis of DEMs in positive ion mode. (**H**): Heatmap of clustering analysis of DEMs in negative ion mode.

The principal component analysis (PCA) of the metabolomic data for *A. oxytropis* OW 7.8 and Δ*swnN* is shown in Figure 10A,B, indicating differences in metabolites between Δ*swnN* and *A. oxytropis* OW 7.8. In positive ion mode, the most abundant metabolites were lipids and lipid-like molecules (30.04%), followed by organic acids and derivatives (23.99%), and organoheterocyclic compounds (14.48%). In negative ion mode, the most abundant metabolites were lipids and lipid-like molecules (40.84%), followed by organic acids and derivatives (20.30%), nucleosides, nucleotides, and analogues (10.89%), and alkaloids and derivatives (9.90%) (Figure 10C,D,G,H). In positive ion mode, 733 DEMs were identified, with 258 up-regulated and 204 down-regulated. In negative ion mode, 271 DEMs were identified, with 172 up-regulated and 99 down-regulated (Table 2).

KEGG enrichment analysis was performed on 733 differential metabolites, and enriched into 57 metabolic groups, including 50 DEMs for metabolic pathways, 29 DEMs for various antibiotic, 20 DEMs for secondary metabolites, 19 DEMs for amino acids, 11 DEMs for 2-oxocarboxylic acid metabolism (Figure 10F). Five DEMs involved in SW synthesis were identified with *L*-glutamate, α-ketoglutaric acid, and *L*-proline up-regulated, while PA and 2-aminoadipic acid were down-regulated (Figure 10E).

### 2.10. Expression of sac, P5CR, and SWN Cluster Genes in A. oxytropis OW 7.8 and ΔswnN

**Figure 11:**
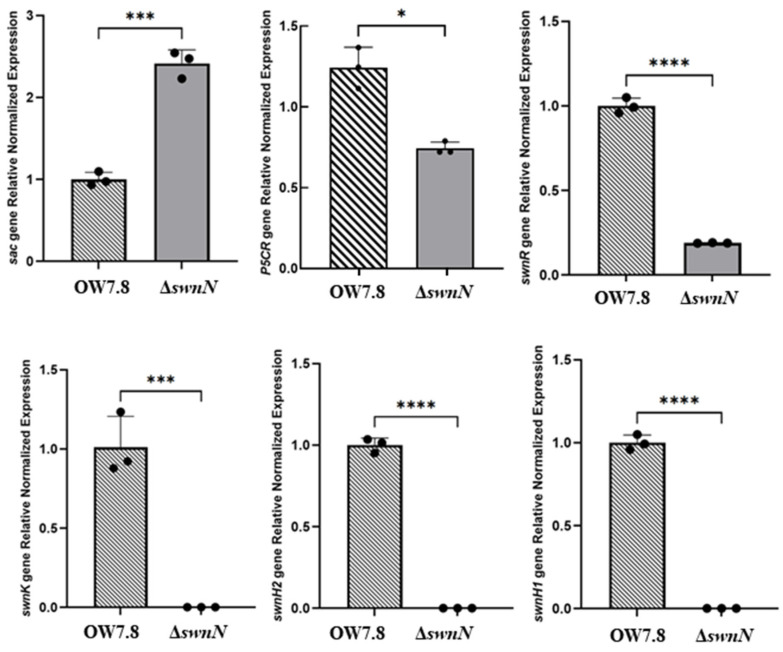
RT-qPCR detection of SW synthesis-related gene expression levels in *A. oxytropis* OW 7.8 and Δ*swnN*. Note: The x-axis represents genes; the y-axis represents relative expression levels. Error bars indicate the standard error of the mean (n = 3), with (*) *p* < 0.05, (***) *p* < 0.001, and (****) *p* < 0.0001.

RT-qPCR results indicated that the relative expression levels of the *sac* gene in Δ*swnN* are significantly increased compared to *A. oxytropis* OW 7.8. Conversely, the relative expression levels of the *P5CR*, *swnH1*, *swnH2*, *swnK*, and *swnR* genes significantly decreased (Figure 11).

### 2.11. SW Synthesis Pathway in A. oxytropis OW 7.8

**Figure 12:**
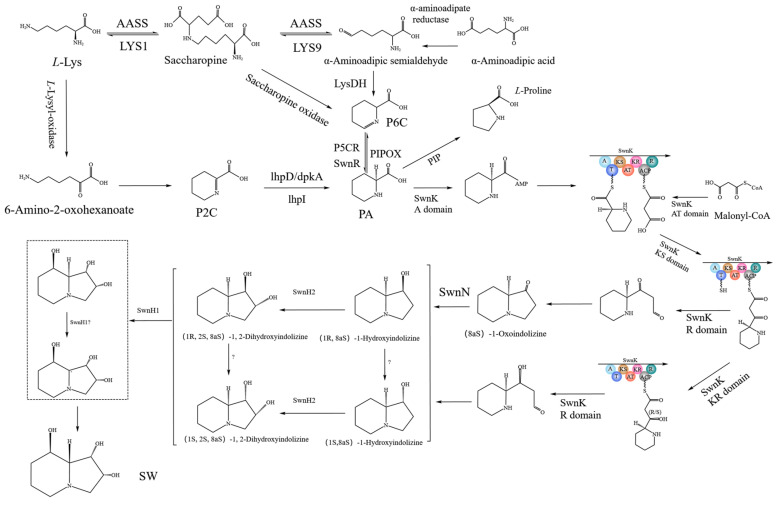
SW synthesis pathway in *A. oxytropis* OW 7.8. Note: LYS1/LYS9: Saccharopine reductase; AASS: α-Aminoadipic semialdehyde synthase; lysDH: Lysine dehydrogenase; PIPOX: *L*-Pipecolic acid oxidase/proline oxidase; P5CR: Pyrroline-5-carboxylate reductase; AAR: α-Aminoadipic acid reductase; *L*-Lysine-oxidase: *L*-Lysine oxidase; dpkA/lhpD/lhpI: 1-Piperideine-2-carboxylate reductase; PIP: Proline iminopeptidase.

The predicted SW biosynthesis pathway in *A. oxytropis* OW 7.8 is shown in Figure 12, starting from *L*-lysine artificially. The Sac enzyme catalyzes the synthesis of saccharopine from *L*-Lysine. Saccharopine is reduced to α-aminoadipic semialdehyde (α-aminoadipic semialdehyde is also formed from α-aminoadipic acid catalyzed by α-aminoadipate reductase). α-Aminoadipic semialdehyde cyclizes to form P6C (P6C is also formed from saccharopine catalyzed by saccharopine oxidase). P6C is then catalyzed by the P5CR enzyme or SwnR enzyme to form *L*-PA. There might be an alternative pathway synthesizing *L*-PA, in which *L*-lysine is converted to 6-amino-2-oxohexanoate catalyzed by *L*-lysyl-alpha-oxidase. 6-Amino-2-oxohexanoate isomerizes to form P2C, which is subsequently catalyzed by the enzymes lhpD/dpkA/lhpI to produce *L*-PA.

*L*-PA was converted to (8aS)-1-oxoindolizine, (1R,8aS)-1-hydroxyindolizine, or (1S,8aS)-1-hydroxyindolizine by the action of multifunctional SwnK protein. Subsequently, (8aS)-1-oxoindolizine was synthesized form (8aS)-1-hydroxyindolizine by SwnN enzyme. (1R,2S,8aS)-1,2-dihydroxyindolizine was synthesized from (1R,8aS)-1-hydroxyindolizine by the action of the SwnH2 enzyme, (1S,2S,8aS)-1,2-dihydroxyindolizine was synthesized from (1S,8aS)-1-hydroxyindolizine by the action of the SwnH2 enzyme. Finally, (1S,2S,8R,8aR)-1,2,8-octahydroindolizinetriol (SW) was synthesized from (1R,2S,8aS)-1,2-dihydroxyindolizine or (1S,2S,8aS)-1,2-dihydroxyindolizine catalyzed by SwnH1 enzyme.

## 3. Discussion

The nucleotide sequence identity of *swnN* gene between *A. oxytropis* OW 7.8 and *A. oxytropis* Raft River (KY365741.1) was 99.91% indicating a close phylogenetic relationship, with one synonymous mutation (at 1053, C in OW 7.8 and G in Raft River, both encoding threonine). The identity of SwnN amino acid sequence was 100% between *A. oxytropis* OW 7.8 and *A. oxytropis* Raft River, while the identity between *A. oxytropis* OW 7.8 and *M. robertsii* ARSEF 2575 was 84.03%. The phylogenetic relationship of SwnN protein (Figure 2B) in SW-producing fungi is consistent with that in the fungi reported by Neyaz et al. [32].

The endophytic fungus *A. oxytropis* OW 7.8 exhibits a very slow growth rate and long growing cycle in vitro, with a very low homologous recombination rate, resulting in few transformants after long-term exploration. In this study, the *swnN* gene of *A.oxytropis* OW 7.8 was knocked out for the first time, and it was found that the SW levels in mycelia of Δ*swnN* were always lower than that in *A. oxytropis* OW 7.8 at different cultivation times. For example, SW was not detected in Δ*swnN* cultured for 20 days, and it was detected in trace amounts in the Δ*swnN* strain cultured for 35 d. SW was detected again in the gene function complementation strain Δ*swnN*/*swnN*, indicating that the function of the *swnN* gene promotes the synthesis of SW in fungi which is consistent with the conclusion in *M. robertsii*.

Compared with *A. oxytropis* OW7.8, the expressions of genes including *P5CR*, *swnR*, *swnK*, *swnH2*, and *swnH1* were all down-regulated in the transcriptome of Δ*swnN*. RT-qPCR results for *A. oxytropis* OW 7.8 and Δ*swnN* were consistent with the data of transcriptome analysis. The levels of PA and α-aminoadipic acid were down-regulated, and *L*-proline was up-regulated in the metabolome of Δ*swnN*. The SW levels in mycelia of Δ*swnN* were always lower than that in *A. oxytropis* OW 7.8. The PA levels were down-regulated, consistent with down-regulated gene expressions of *P5CR* and *swnR* encoding enzymes involved in PA synthesis. Down-regulation of α-aminocaproic acid and up-regulation of *L*-proline might also contribute to decrease of PA levels. Knockout of *swnN* gene might result in the accumulation of substrate of SwnN enzyme, which could decrease the expressions of *swnK* gene due to negative feedback regulation. SW could not be synthesized through the 1-oxoindolizine branch after knocking out the *swnN* gene, but it could be synthesized through the 1-hydroxyindolizine branch. The synthesis of 1-hydroxyindolizine might be decreased due to down-regulation of the *swnK* gene. Since the expressions of downstream *swnH2* gene and *swnH1* gene were also determined showing down-regulation, the SW levels were greatly decreased, finally, in Δ*swnN*.

Significant gene expression changes occurred in Δ*swnN* in multiple metabolic groups through GO enrichment, including biological processes, cell components, and molecular functions. Transcriptome and metabolome joint analysis of *A. oxytropis* OW7.8 and Δ*swnN* showed significant changes in levels of gene expression and metabolites in some metabolic groups such as biosynthesis of secondary metabolites, biosynthesis of amino acids, 2-oxocarbonyl acid metabolism, lysine biosynthesis, and lysine degradation. We postulated that the function of SwnN protein was involved in both SW biosynthesis and other physiological and biochemical reactions in *A. oxytropis* OW7.8. Therefore, the colonies and mycelia morphology, and growth rate changed in Δ*swnN.*

Our previously predicted SW biosynthesis pathway in *A. oxytropis* OW 7.8 included P6C and P2C branches [36]. In this study, the P6C branch in *A. oxytropis* OW 7.8 was refined, predicting that α-aminoadipic semialdehyde can also be produced from α-aminoadipic acid catalyzed by α-aminoadipate reductase, and P6C can also be formed from saccharopine catalyzed by saccharopine oxidase. The pathway from *L*-PA to SW was also refined, including the conversion of *L*-PA to (8aS)-1-indolizidinone, (1R,8aS)-1-hydroxyindolizine, or (1S,8aS)-1-hydroxyindolizine by action of the SwnK protein, the conversion from (8aS)-1-oxoindolizine to (8aS)-1-hydroxyindolizine catalyzed by the SwnN enzyme, the conversion from (1R,8aS)-1-hydroxyindolizine and (1S,8aS)-1-hydroxyindolizine to (1R,2S,8aS)-1,2-dihydroxyindolizine, (1S,2S,8aS)-1,2-dihydroxyindolizine catalyzed by the SwnH2 enzyme, the conversion of (1R,2S,8aS)-1,2-dihydroxyindolizine and (1S,2S,8aS)-1,2-dihydroxyindolizine to SW catalyzed by the SwnH1 enzyme.

## 4. Materials and Methods

### 4.1. Strain

*A. oxytropis* OW 7.8 was isolated by our research group from *O. glabra* collected in Wushen Banner, Ordos city, Inner Mongolia, China (108°52′ E, 38°36′ N, elevation 1291 m) [17]. The mycelia were cultured on potato dextrose agar (PDA) media (fresh peeled potatoes 200 g, glucose 20 g, agarose 15 g, diluted to 1000 mL) at 25 °C.

### 4.2. Extraction of Genomic DNA and Identification of swnN Gene from A. oxytropis OW 7.8

The genomic DNA of *A. oxytropis* OW 7.8 was extracted (Plant Genomic DNA Kit, Tiangen, Beijing, China). The quality of the DNA was verified by 1% agarose gel electrophoresis. Primers NupF (5′-CTGGCTAGCTGCATATGCAGCAG-3′) and NdownR (5′-CGTGGCAGTTGATGACTGGG-3′) were designed based on the genomic data of *A. oxytropis* OW 7.8 to amplify the *swnN* gene. The PCR program was as follows: 94 °C for 3 min; 94 °C for 30 s, 58 °C for 30 s, 72 °C for 70 s, repeated for 29 cycles; and a final extension at 72 °C for 10 min. The PCR products were detected by 1% agarose gel electrophoresis, purified (SanPrep Column PCR Product Purification Kit, Sangon Biotech, Shanghai, China), and then sequenced (Sangon Biotech).

### 4.3. Total RNA Extraction and swnN cDNA Cloning of A. oxytropis OW 7.8

Total RNA of *A. oxytropis* OW 7.8 was extracted (the OminiPlant RNA Kit, CWBIO, Shanghai, China) and reverse-transcribed to synthesize cDNA. The *swnN* cDNA was amplified with primers NF/NR (5′-CCTCGACTCTAGAGGATCCATGGTTGTCGTTGCTGTCGCC-3′; 5′- CCTCGCCCTTGCTCACCATCACAAGCTCCCTGTGATCAAGAT-3′). The PCR program was as follows: 98 °C for 3 min; 98 °C for 30 s, 58 °C for 30 s, 72 °C for 60 s, repeated for 29 cycles; and a final extension at 72 °C for 10 min. The PCR products were detected by 1.0% agarose gel electrophoresis, purified (SanPrep Column PCR Product Purification Kit, Sangon Biotech, Shanghai, China), and then sequenced (Sangon Biotech, Shanghai, China).

### 4.4. Construction of Vectors

#### 4.4.1. Construction of the *swnN* Gene Knockout Vector

**Figure 13:**
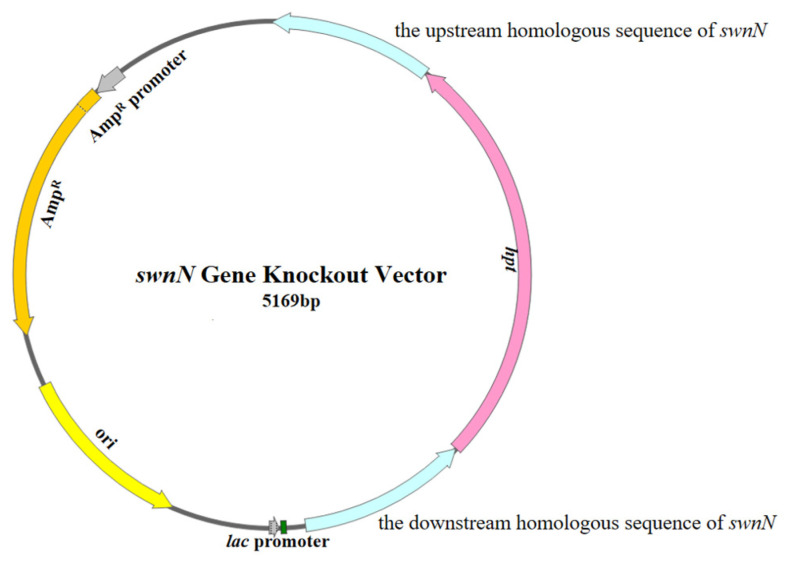
Diagram of the *swnN* gene knockout vector structure.

The upstream and downstream homologous sequences of the *swnN* were amplified using the genomic DNA of *A. oxytropis* OW 7.8 as a template and primers upF/upR (5′-CTGGCTAGCTGCATATGCAGCAG-3′; 5′-CTGGCTAGCTGCATATGCAGCAG-3′) and downF/downR (5′-ATAGAGTAGATGCCGACCGCGGGTTCGCCATCCATGGAGGCCTCAT-3′; 5′-CGTGGCAGTTGATGACTGGG-3′). The *hpt* 5′ and 3′ end sequences were amplified using the pCB1003 plasmid as a template and primers HYGF/hygR (5′-GGCTTGGCTGGAGCTAGTGGAGGTCAA-3′; 5′-GTATTGACCGATTCCTTGCGGTCCGAA-3′) and hygF/HYGR (5′-GATGTAGGAGGGCGTGGATATGTCCT-3′; 5′-GAACCCGCGGTCGGCATCTACTCTAT-3′). The upstream and downstream homologous sequences of the *swnN* were linked to the *hpt* gene on both sides using the split-marker technique to construct the *swnN* gene knockout cassette, and this cassette was then ligated into the pMD-19T vector (Takara, Beijing, China) using TA cloning to make up the *swnN* gene knockout vector (Figure 13).

#### 4.4.2. Construction of the *swnN* Gene Complementation Vector

The pBARGPE1-EGFP vector (MIAO LING BIOLOGY, Wuhan, China) was digested with *Eco*RI (Takara, Beijing, China) and *Bam*HI (Takara, Beijing, China), reaction at 37 °C for 4 h, enzyme inactivation at 60 °C for 15 min. The *swnN* cDNA with homologous regions of the vector was ligated into the above linearized pBARGPE1-EGFP vector by seamless cloning. The *bar* was used as a selection marker, with PgpdA as the promoter and TtrpC as the terminator, to drive the expression of *swnN* cDNA.

### 4.5. Sensitivity Test of A. oxytropis OW 7.8 to Hygromycin B

The mycelia of *A. oxytropis* OW 7.8 were inoculated on PDA media containing 0 μg/mL, 0.8 μg/mL, 0.9 μg/mL, 1 μg/mL,2 μg/mL, and 3 μg/mL of Hyg B, respectively. The cultures were incubated in the dark at 25 °C for 20 days. The growth of colonies was observed, and an appropriate concentration of Hyg B was chosen to add to the media for *swnN* gene knockout transformant screening.

### 4.6. Preparation and Transformation of A. oxytropis OW 7.8 Protoplasts

Young mycelia of OW7.8 were inoculated in PDB media (50 μg/mL Amp) shaking at 25 °C, 200 rpm for 7 days. Protoplasts from mycelia of *A. oxytropis* OW 7.8 were prepared according to the method described by Hu et al. [40]. The *swnN* gene knockout vector was then transformed into protoplast of *A. oxytropis* OW 7.8 mediated by PEG8000 [40].

### 4.7. Screening and Identification of Gene Knockout Transformants

**Figure 14:**
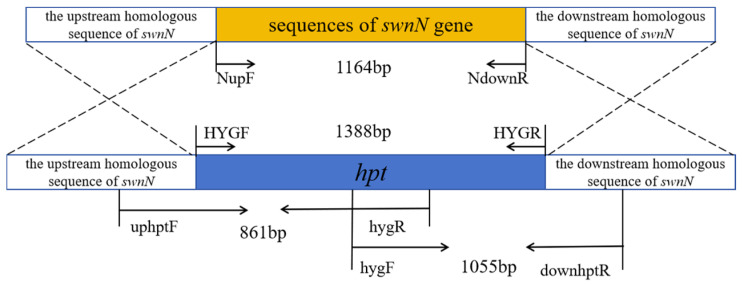
Identification figure for *swnN* gene knockout transformants.

The *swnN* gene knockout transformants were cultured on TB_3_ (0.3% yeast extract, 0.3% acid hydrolyzed casein, 20% sucrose) regeneration media (bottom layer: 50 μg/mL Amp and 1 μg/mL Hyg B; top layer: 50 μg/mL Amp and 2 μg/mL Hyg B) at 25 °C to cultivate. Single colony was transferred to PDA media (2 μg/mL Hyg B) for culture. Transformants resistant to Hyg B were screened and subjected to PCR analysis. The sequence of upstream homologous and *hpt* was amplified with primers uphygF/hygR (5′-GCACAGGGCCATCGTACTATCC-3′/5′-GTATTGACCGATTCCTTGCGGTCCGAA-3′). The sequence of *hpt* and downstream homologous sequence was amplified with primers hygF/downhygR (5′-GATGTAGGAGGGCGTGGATATGTCCT-3′/5′-GCGCATGACTCAACATTGAGAG-3′). The sequence of *hpt* was amplified with primers HYGF/HYGR (5′-GGCTTGGCTGGAGCTAGTGGAGGTCAA-3′/5′-GAACCCGCGGTCGGCATCTACTCTAT-3′) (Figure 14). All PCR products were sequenced for verification (Sangon Biotech, Shanghai, China).

### 4.8. Sensitivity Test of ΔswnN to Glufosinate

The mycelia of Δ*swnN* were inoculated on PDA media containing 300 μg/mL, 400 μg/mL, and 500 μg/mL of Gla, respectively. The cultures were incubated in the dark at 25 °C for 20 days. The growth of colonies was observed, and an appropriate concentration of Gla was chosen to add to the media for the *swnN* gene complementation transformant screening.

### 4.9. Preparation and Transformation of ΔswnN Protoplasts

Young mycelia of Δ*swnN* were inoculated in PDB media (50 μg/mL Amp, 2 μg/mL Hyg B) shaking at 25 °C, 200 rpm for 14 days. Protoplasts were prepared according to the method described by Hu et al. [40]. The *swnN* gene complementation vector was then transformed into protoplast of Δ*swnN* mediated by PEG8000 [40].

### 4.10. Screening and Identification of Gene Complementation Transformants

**Figure 15:**
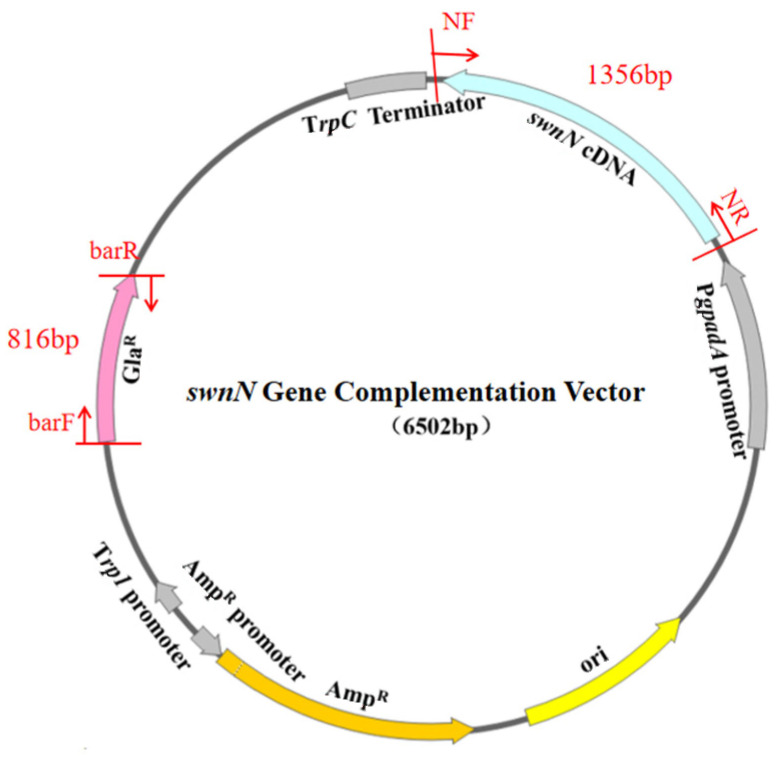
Diagram of the *swnN* gene complementation vector structure.

The *swnN* gene complementation transformants were cultured on TB_3_ regeneration media (bottom layer: 50 μg/mL Amp and 1 μg/mL Hyg B; top layer: 50 μg/mL Amp, 2 μg/mL Hyg B, and 500 μg/mL Gla) at 25 °C. Single colony was transferred to PDA media (50 μg/mL Amp, 2 μg/mL Hyg B, and 500 μg/mL Gla) for culture. The *swnN* gene complementation transformants resistant to Gla were screened and subjected to PCR analysis. The sequence of *bar* gene was amplified with primers barF/barR (5′-ATTAGCAGACAGGAACGAGGACA-3′/5′-CATCGCAAGACCGGCAAC-3′), and the *swnN* cDNA was amplified with primers NF/NR (5′-GTCAGGGTGGTCACGAGGG-3′/5′-CAAGGTCGTTGCGTCAGTCC-3′). All PCR products were sequenced for verification (Sangon Biotech, Shanghai, China). The placement of primers and expected size of PCR products are shown in Figure 15.

### 4.11. Colony and Mycelia Morphology

The colony morphology of *A. oxytropis* OW 7.8 and Δ*swnN* after 20 days of cultivation was observed. The mycelia morphology of *A. oxytropis* OW 7.8 and Δ*swnN* was observed under a scanning electron microscope (SU81003.0 kV × 30, Wuhan Servicebio Technology, Wuhan, China).

### 4.12. Extraction and Detection of SW in Mycelia of A. oxytropis OW 7.8, ΔswnN, and ΔswnN/swnN

Mycelia of *A. oxytropis* OW 7.8, Δ*swnN* and Δ*swnN/swnN* cultured for 20 days were used for SW extraction by acetic acid–chloroform solution, purification by cation exchange resin, and elution with 1 mol/L ammonia solution. The SW levels in the mycelia were determined by HPLC-MS, with three replicates for each sample [41]. Data were analyzed by one-way ANOVA in GraphPad Prism 9.5 software. The chromatographic conditions were as follows: mobile phase, 5% methanol and 20 mmol/L ammonium acetate; flow rate, 0.4 mL/min; column temperature, 30 °C. MS conditions were as follows: positive ion 156; negative ion 70; IonSpray voltage (IS), 30 V.

### 4.13. Transcriptome Analysis of A. oxytropis OW 7.8 and ΔswnN

Mycelia of *A. oxytropis* OW 7.8 and Δ*swnN* strains (fresh weight 0.1–0.3 g) cultured for 20 days were rapidly frozen with liquid nitrogen in cryovials (1–3 min), and then delivered on dry ice with three replicates for each sample. Transcriptome sequencing was performed on the Illumina NovaSeq 6000 platform (Novogene, Beijing, China). The data were analyzed using DESeq2 (1.20.0) [42] and clusterProfiler (3.8.1) [43] package in R (4.1.0). The differentially expressed genes (DEGs|log_2_(Fold Change)|≥1 and *p*-value ≤ 0.05) [42,44] were subjected to functional enrichment analysis with GO and KEGG.

### 4.14. Metabolome Detection and Analysis of A. oxytropis OW 7.8 and ΔswnN

Mycelia of *A. oxytropis* OW 7.8 and Δ*swnN* (fresh weight 0.1–0.3 g) cultured for 20 days were rapidly frozen with liquid nitrogen in cryovials (1–3 min), and then delivered on dry ice with five replicates for each sample. Metabolome was performed using LC-MS (Novogene, Beijing, China). The data were analyzed using Compound Discoverer (3.1) package in R [45]. The differential expressed metabolites (DEMs VIP > 1, |log_2_(Fold Change)| ≥ 1 and *p*-value < 0.05) [46,47] were subjected to functional enrichment analysis with KEGG.

### 4.15. RT-qPCR Detection of Genes Closely Related to SW Synthesis in A. oxytropis OW 7.8 and ΔswnN

The cDNAs of *A. oxytropis* OW 7.8 and Δ*swnN* were used as templates with actin as internal reference gene [48] to conduct RT-qPCR to amplify six differentially expressed genes closely related to SW synthesis. The PCR program was as follows: 95 °C for 2 min; 95 °C for 5 s, 60 °C for 30 s, repeated for 29 cycles, 95 °C for 20 s, 65 °C for 1 min,95 °C for 20 s. The RT-qPCR for six genes of *sac* (QsacF/QsacR 5′-CTGCTGCTCGGTGCTGGATTC-3′; 5′-CTAGACTGATGGCGTTGGTGTTGG-3′), *P5CR* (QP5CRF/QP5CRR 5′-TAGCAATAATGGGCGGCGTGATG-3′; 5′-GGCGATGAAGTTGGAGAGGTTGG-3′), *swnR* (QswnRF/QswnRR 5′-TTCTACTTTGCCACACACGAACCC-3′; 5′-ATAGTCAGCCAACCAGCCAATGC-3′), *swnK* (QswnKF/QswnKR 5′-GACCGCTTGCTCGCCTGTG-3′; 5′-CTCGTCAACTCGTCCAACACTTCC-3′), *swnH1* (QswnH1F/QswnH1R 5′-TTGCTTTGCGGAGATGGAACCAG-3′; 5′-CGGAGTGTGCCTGAGATGAAGAAG-3′), and *swnH2* (QswnH2F/QswnH2R 5′-CATCTGCTCCTCGCTTGCTACC-3′; 5′-CAGGACAACGCCTCCATCTCTTTC-3′) were performed with the 2^(−ΔΔCt) method (ΔCt = Ct(*gene*) − Ct(*actin*), ΔΔCt = ΔCt(∆*swnN*) − ΔCt(OW 7.8)). All statistical analysis was conducted by GraphPad Prism 9.5.0 software with one-way ANOVA for each group of samples.

## 5. Conclusions

The *swnN* gene of endophytic fungus *Alternaria oxytropis* OW 7.8 isolated from *Oxytropis glabra* was cloned, and the gene knocked out mutant ∆*swnN* was first constructed. The colony morphology of Δ*swnN* differed from that of *A. oxytropis* OW 7.8, appearing creamy yellow, with irregular shapes and a slower growth rate. Compared to *A. oxytropis* OW 7.8, no SW was detected in the mycelia of ∆*swnN* cultured for 20 days, and SW was detected again in the gene function complementation strain Δ*swnN*/*swnN*, indicating that the function *swnN* gene promotes SW biosynthesis. Six DEGs and five DEMs closely associated with SW biosynthesis were identified by analyzing the data of the transcriptome and metabolome of *A. oxytropis* OW 7.8 and Δ*swnN*. The SW biosynthesis pathway in *A. oxytropis* OW 7.8 was hypothesized and refined.

## Figures and Tables

**Table 1:** Members of “SWN” gene cluster and their function prediction [31].

Gene	Encoding Product	Function Prediction
*swnA*	Aminotransferase	Catalyzing the synthesis of Pyrroline-6-carboxylate (P6C) from *L*-Lysine
*swnR*	Dehydrogenase or reductase	Catalyzing the synthesis of *L*-PA from P6C
*swnK*	Multifunctional protein	Catalyzing the synthesis of 1-Oxoindolizidine (or 1-Hydroxyindolizine) from *L*-PA
*swnN*	Dehydrogenase or reductase	Catalyzing the synthesis of 1-Hydroxyindolizine from 1-Oxoindolizidine
*swnH1*	Fe (II)/α-Ketoglutarate-dependent dioxygenase	Catalyzing the synthesis of SW from 1,2-Dihydroxyindolizine
*swnH2*	Fe (II)/α-Ketoglutarate-dependent dioxygenase	Catalyzing the synthesis of 1,2-Dihydroxyindolizine form 1-Hydroxyindolizine
*swnT*	Transmembrane transporter	Transport of SW

**Table 2:** Screening of DEMs.

Screening Mode	Total of Metabolites	Total of DEMs	Up-Regulated	Down-Regulated
Positive	801	462	258	204
Negative	472	271	172	99

## Data Availability

The data presented in this study are available on reasonable request from the corresponding author.
